# Vertically stacked skin-like active-matrix display with ultrahigh aperture ratio

**DOI:** 10.1038/s41377-024-01524-z

**Published:** 2024-07-26

**Authors:** Juntong Li, Yanping Ni, Xiaoli Zhao, Bin Wang, Chuang Xue, Zetong Bi, Cong Zhang, Yongjun Dong, Yanhong Tong, Qingxin Tang, Yichun Liu

**Affiliations:** Key Laboratory of UV-Emitting Materials and Technology of Ministry of Education, Northeast Normal University, Changchun, China

**Keywords:** Photonic devices, Organic LEDs, Displays, Polymers, Lithography

## Abstract

Vertically stacked all-organic active-matrix organic light-emitting diodes are promising candidates for high-quality skin-like displays due to their high aperture ratio, extreme mechanical flexibility, and low-temperature processing ability. However, these displays suffer from process interferences when interconnecting functional layers made of all-organic materials. To overcome this challenge, we present an innovative integration strategy called “discrete preparation-multilayer lamination” based on microelectronic processes. In this strategy, each functional layer was prepared separately on different substrates to avoid chemical and physical damage caused by process interferences. A single interconnect layer was introduced between each vertically stacked functional layer to ensure mechanical compatibility and interconnection. Compared to the previously reported layer-by-layer preparation method, the proposed method eliminates the need for tedious protection via barrier and pixel-defining layer processing steps. Additionally, based on active-matrix display, this strategy allows multiple pixels to collectively display a pattern of “1” with an aperture ratio of 83%. Moreover, the average mobility of full-photolithographic organic thin-film transistors was 1.04 cm^2^ V^−1^ s^−^^1^, ensuring stable and uniform displays. This strategy forms the basis for the construction of vertically stacked active-matrix displays, which should facilitate the commercial development of skin-like displays in wearable electronics.

## Abstract (web summary)

This study proposes the “discrete preparation-multilayer lamination” strategy, which effectively addresses the challenges associated with vertically stacked skin-like AMOLEDs and demonstrates their potential for next-generation wearable displays.

## Graphical abstract

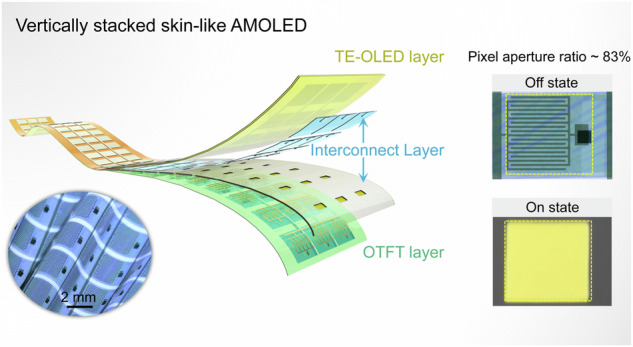

## Introduction

Skin-like displays are critical components of information output in next-generation portable and wearable electronics. Such displays must be highly flexible to maintain intimate and comfortable contact with the human body^1–5^. Developing active-matrix displays comprising organic thin-film transistors (OTFTs) and organic light-emitting diodes (OLEDs) is crucial for achieving high-quality skin-like displays^6–8^. Although skin-like displays can be based on passive- or active-matrix designs depending on the light source^9–11^, the latter is advantageous because it minimizes signal crosstalk and offers better spatial resolution and contrast and a faster response^12,13^. Additionally, all-organic active-matrix OLEDs (AMOLEDs) offer mechanical flexibility and low-temperature processing^14,15^, facilitating the integration of various flexible or elastic materials to fabricate high-flexible skin-like displays^16,17^. Several research groups have reported that integrating OTFT and OLED on flexible substrates produces superior display performance. For example, Zyung et al. reported a flexible AMOLED of white OLEDs driven vertically by OTFTs^18^, achieving a luminance of 180 cd m^−2^ at a current density of 5 mA cm^−^^2^. Yagi et al. introduced a double-layer organic dielectric layer, comprising a low-damage passivation layer and planarization layer, onto the OTFT backplane, producing a full-color flexible AMOLED with a resolution of 80 ppi^19^.

The aperture ratio is the ratio of the active area to the total pixel area and is a key performance indicator for display technology^20–23^. However, fabricating skin-like AMOLED displays with a high aperture ratio remains a tremendous challenge. Almost all reported AMOLED displays are parallel structures consisting of side-by-side OTFTs and OLEDs (see Table S1). Unfortunately, this parallel structure covers part of the pixel area by nonluminous OTFTs and wiring, resulting in low active area, luminance, resolution, and aperture ratio. Therefore, the aperture ratio of previously reported parallel structured AMOLEDs is generally less than 37%^24–30^, and the maximum aperture ratio is only 52%^31^.

The vertical structure is more conducive than the parallel structure for fabricating AMOLEDs with a high aperture ratio^6,32,33^. Vertically stacking OLEDs and OTFTs eliminates obstruction from nonluminous OTFTs and wiring parts, maximizing the active area. In addition, a vertically structured pixel minimizes the occupied area, dramatically improving the display’s resolution and quality. For instance, Jeong et al. demonstrated that the aperture ratio of a vertical pixel structure with three polymer protective layers inserted between the pixel circuits of OLEDs and OTFTs was over 2.5 times that of the parallel pixel structure^33^. Steudel et al. reported a flexible vertically stacked AMOLED on a plastic substrate with a maximum pixel aperture ratio of 75%^32^. Nevertheless, fabricating vertically structured skin-like AMOLEDs is complex because such displays introduce new challenges, including mutual dissolution, mechanical compatibility, and interconnection between functional layers^19^. Existing fabrication strategies still struggle to address these problems simultaneously, resulting in only a few reports of vertically structured AMOLED displays (Table S1). Moreover, all such displays are fabricated on glass or thick plastic substrates, limiting the inherent mechanical flexibility of organic materials and preventing intimate skin contact.

Herein, we propose a novel strategy to demonstrate vertically stacked skin-like active-matrix displays. Specifically, we separately prepare each conformable functional layer (top-emitting OLED (TE-OLED)/interconnect layer/all-photolithographic OTFTs) and then laminate them together, thereby avoiding chemical and physical damage and ensuring mechanical compatibility and interconnection between functional layers. Using this versatile strategy, the aperture ratio of a pixel reaches 83%, which is superior to all the reported AMOLEDs. The OLED device maintains 80% of its initial current density even when the bending radius is as small as 2.5 mm, and the all-photolithographic OTFTs maintain 92% of the initial mobility value after 10,000 folding cycles. More strikingly, all-organic AMOLEDs have outstanding mechanical flexibility and conformability, allowing them to operate normally, even on three-dimensional (3D) curved objects or skin. This “discrete preparation-multilayer lamination” strategy offers a universal platform for constructing high-aperture-ratio AMOLEDs, maximizes the advantages of organic materials (e.g., mechanical flexibility and low-temperature processing), and demonstrates a strong potential for the development of skin-like displays in next-generation wearable electronics.

## Results

### Design and fabrication

**Fig. 1:**
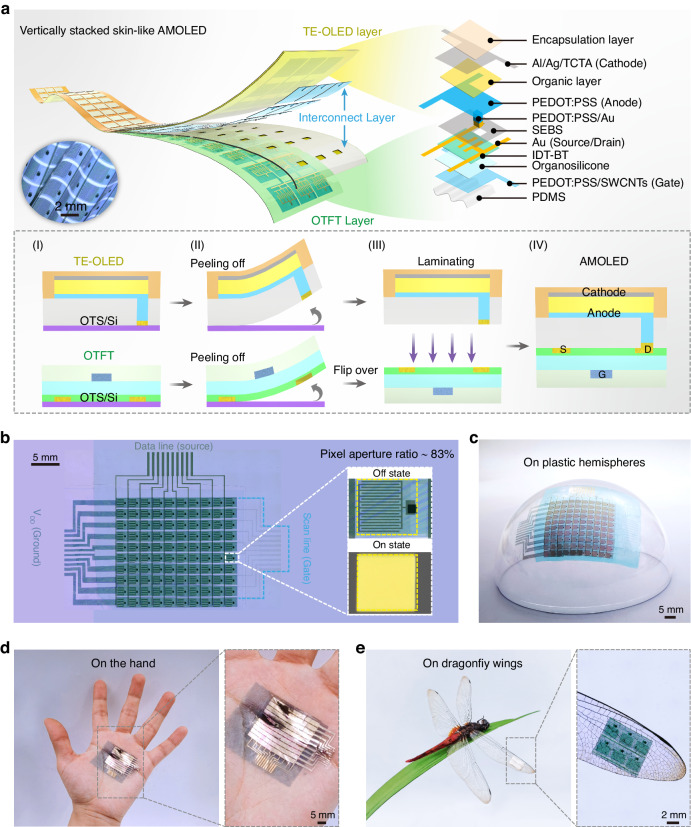
**Constructing a vertically stacked skin-like AMOLED display array**. **a** Schematics of skin-like AMOLED device and main fabrication scheme (bottom inset). **b** 3D optical microscope image. Illustrations are digital photographs and microscope images of individual pixels. **c** Photograph of a skin-like AMOLED array adhered to a transparent plastic hemisphere. **d** Photographs of the array adhered onto a human palm. **e** Photographs and 3D optical microscope images of the array conformed onto a dragonfly wing

To fabricate vertically stacked skin-like active-matrix displays, we developed a “discrete preparation-multilayer lamination” strategy. Figure 1a shows the structural diagram, detailed composition, and the main fabrication scheme of the display (see the Experimental Section and Fig. S1 for details). The display consists of three parts: the conformable TE-OLED (top layer), the conformable interconnect layer (middle layer), and the conformable all-photolithographic OTFT (bottom layer). Initially, the interconnect layer as a “connecting link” was patterned by photolithography combined with oxygen plasma treatment on an octadecyl trichlorosilane-modified (OTS-modified) silicon wafer (Fig. 1aI). Pre-modifying the silicon wafer with a hydrophobic OTS monolayer serves to significantly reduce the intermolecular force (Fig. S2). Poly(styreneethylene-butylene-styrene) (SEBS) and poly(3,4-ethylenedioxythiophene): polystyrene sulfonate (PEDOT:PSS)/Au serve as electrical isolation and electrical connection between the upper OLED and the lower OTFT of the display, respectively. The inherent dielectric and viscoelasticity of SEBS allow it not only to prevent current leakage from the upper and lower functional layers but also to ensure a tight bond between layers^34,35^ (Fig. S3). To verify that the SEBS interconnect layers are in close contact with the OTFTs, we measured their adhesion (see Fig. S4). In addition, we compared other commonly used interconnect layers, including polyvinyl alcohol (PVA) and polydimethylsiloxane (PDMS). The results show that the adhesion of PVA, PDMS, and SEBS to OTFTs is 1.8 N m^−^^1^, 4.4 N m^−1^, and 14.5 N m^−^^1^, respectively. Note that the adhesion of SEBS is about eight times that of PVA and about three times that of PDMS. High-fluidity PEDOT:PSS and high-conductivity Au not only guarantee adequate filling holes but also ensure good electrical conductivity between the upper OLED anode and the lower OTFT drain electrode. Subsequently, a TE-OLED was deposited on the interconnect layer and encapsulated in a transparent photopolymer (NOA63) (Fig. 1aI). NOA63 has greater than 98% transmittance across the visible spectrum (380–780 nm) (Fig. S5), rendering it an exceptional material for constructing TE-OLEDs. NOA63 acts as an encapsulation layer to prevent moisture and oxygen from damaging the OLED^36^ and serves as a support layer to allow the OLED and interconnect layer to more easily peel off the OTS-modified silicon wafer (Fig. 1aII). Finally, the all-photolithographic OTFT with a bottom-gate top-contact configuration was peeled from the OTS-modified silicon wafer. Subsequently, the all-photolithographic OTFT was laminated with the OLED and interconnect layer under a microscope to form skin-like active-matrix displays (Fig. 1aIII, aIV)^37^. The 3D optical microscope image in the left panel of Fig. 1a shows the extreme flexibility of a completed display, and the right panel of Fig. 1a shows its detailed composition. Our experiments introduced no solvents or water during the peeling process, and no additional adhesives were used during the lamination process. This all-dry fabrication avoids mutual dissolution and interface pollution, providing a clean, complete contact interface for fabricating high-quality vertically stacked skin-like displays.

Compared with existing active-matrix display fabrication strategies, the proposed strategy has several advantages: (i) in contrast with the parallel AMOLED preparation strategies, we separately prepared each conformable functional layer (TE-OLED/interconnect layer/all-photolithographic OTFT) and then vertically laminated them together. This strategy significantly improves the aperture ratio. Figure 1b shows 3D optical microscope images of the fabricated vertically stacked AMOLED array. By linking the interconnect layer, the OLED array becomes intimately and vertically stacked on an all-photolithographic OTFT array. The entire array is complete and has no deletions or cracks. The aperture ratio of a pixel is as high as 83%, and the aperture ratio of the display array reaches 71%, both of which surpass the results of all previously reported flexible AMOLED displays. (ii) Unlike conventional layer-by-layer deposition techniques, this strategy eliminates chemical (i.e., various solvents) or physical (i.e., etching and heating) damage to fragile organic materials during fabrication, which is essential for fabricating reproducible, high-quality vertically stacked skin-like displays. In addition, this strategy can integrate modern microelectronic techniques such as photolithography and etching into the skin display, allowing mass production of highly integrated, high-resolution skin-like displays^38,39^. (iii) In contrast with the layer-by-layer preparation on rigid or flexible substrates, all functional layers are stripped from rigid substrates, liberating them from any mechanical limitations imposed by these substrates. Thus, the proposed display has outstanding mechanical flexibility and good conformability. To verify these characteristics, we attached the display array to a 3D transparent plastic hemisphere and a human palm, as shown in Fig. 1c, d, respectively. Benefiting from its good mechanical flexibility, the display array adhered conformably to the hemispherical surface without bubbles and wrinkles. Owing to strong interlayer bonding, the transfer and lamination of the functional layers are seamless, with no dislocation or delamination. Figure 1d shows that the display adheres tightly to a human hand, resembling skin. Moreover, the display is extremely light (65 g m^−2^; Fig. S6), which is even less than the areal density of A4 paper (70 g m^−^^2^). Its ultra-lightness allows the device to conform to the wing of a dragonfly without affecting the wing quiver (Fig. 1e). These results fully testify that this reliable fabrication strategy enables the design of high-aperture-ratio vertically stacked skin-like displays, showing promise for fabricating mass-produced high-performance portable and wearable electronics.

### Interconnect layer

**Fig. 2:**
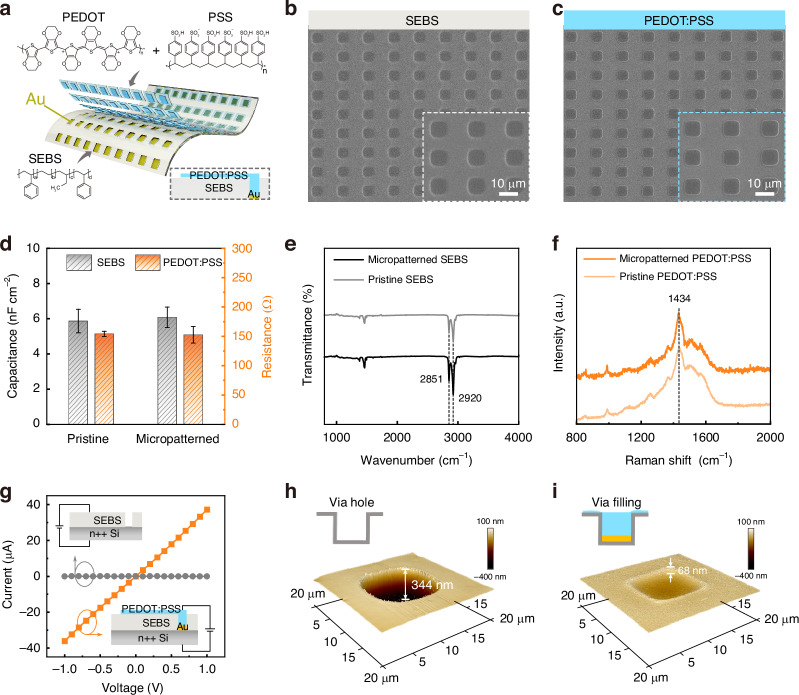
**Conformal interconnect layers**. **a** Device schematics and molecular structures of SEBS and PEDOT:PSS. The interconnect layer is composed of the post-fill hole layer (PEDOT:PSS), the pre-fill hole layer (Au) structure, and the via-hole layer (SEBS). **b**, **c** SEM images of micropatterned via-hole materials (SEBS) and fill-hole materials (PEDOT:PSS), respectively. **d** Electrical properties before and after patterning of via-hole and fill-hole materials. **e** FTIR spectroscopy of via-hole materials and **f** Raman spectroscopy of fill-hole materials. **g** Conductivity before and after hole filling. **h**, **i** 3D AFM image (20 µm × 20 µm) of the via-hole layer. The thickness before (344 nm) and after (68 nm) hole filling, respectively

The interconnect layer is the core component in AMOLED and plays a vital connecting role in vertically stacked circuits^39,40^. Figure 2a shows the structural diagrams of the interconnect layer and the molecular formulas of SEBS and PEDOT:PSS. The intrinsic weak intermolecular forces of organic materials hinder their use in photolithography and cause chemical damage (from solvents such as developer and remover) or physical damage (from etching or heating)^1,18^. However, large-scale photolithography has proven suitable for large-scale, high-precision, and high-integration production, providing a significant advantage. To obtain high-precision and large-scale via holes and filling holes, we devised a nondestructive approach that combines photolithography with plasma etching, thereby enabling the precise definition of SEBS and PEDOT:PSS micropatterns. The SEBS film was initially spin-coated onto an OTS-modified silicon wafer and cured, then the size and location of the hole were defined using photolithography, and the unwanted SEBS was etched by oxygen plasma to form a via hole. Before spin-coating photoresist, PVA and diketopyrrolopyrrole thieno[3,2-b]thiophene (DPPT-TT) membranes were spin-coated on the SEBS to serve as protective layers to avoid solvent-induced SEBS dissolution or swelling during photolithography and high-precision, and large-scale patterning. To achieve a reliable electrical connection between the interconnect layer and the drain electrode of all-photolithographic OTFTs, we used Au in the pre-fill hole and PEDOT:PSS in the post-fill hole, which were meticulously micropatterned following the same methodology used for SEBS (Fig. S7).

Figure 2b, c shows the concave SEBS and the convex PEDOT:PSS micropattern array, respectively. Their micropatterns remain regular and uniform on a micron scale. The accuracy is much greater than commonly used perforation techniques such as laser ablation or mechanical perforation. In addition, their electrical properties barely change upon micropatterning. As shown in Fig. 2d, the capacitance of SEBS increased by 3.6%, and the conductivity of PEDOT decreased by 1%, which is almost negligible. To further elucidate the changes in the chemical composition of SEBS during the micropatterning process, Fourier transform infrared spectra of SEBS were acquired before and after micropatterning (Fig. 2e). After SEBS micropatterning, the intensity of the peaks attributed to alkyl stretching vibrations at 2851 cm^−1^ and 2920 cm^−1^ remained essentially constant, indicating that the micropatterning process did not structurally change the SEBS^41^. Figure S8 shows an atomic force microscopy (AFM) image of the SEBS before and after fabricating the via holes. We used Raman spectroscopy to monitor the surface chemical bond of PEDOT:PSS during micropatterning. Figure 2f shows that after PEDOT:PSS micropatterning, the intensity of the vibration peak caused by the C=C symmetric stretching of PEDOT:PSS at 1434 cm^−1^ remained unchanged, indicating that the micropatterning process does not damage PEDOT:PSS^42^. These experimental results confirm that the proposed photolithographic approach is compatible with organic materials, facilitating nondestructive and high-precision preparation of the interconnect layer. To further demonstrate the electrical isolation and electrical connection of the interconnect layer, we acquired current–voltage (*I*–*V*) and AFM data via holes and filling holes, as shown in Fig. 2g–i. The location filled by PEDOT:PSS/Au exhibits high electrical conductivity with a resistance as low as 28 kΩ, while the other location maintains its inherent dielectric characteristic with a resistance as high as 375 GΩ (Fig. 2g). In addition, 3D AFM images further confirm that the hole is filled with PEDOT:PSS/Au, showing a depth reduction from 344 nm to 68 nm (Fig. 2h, i). These results demonstrate that the photolithographic interconnect layer should work properly, providing a solid foundation for the subsequent integration of vertically stacked devices.

### Top-emitting OLEDs

**Fig. 3:**
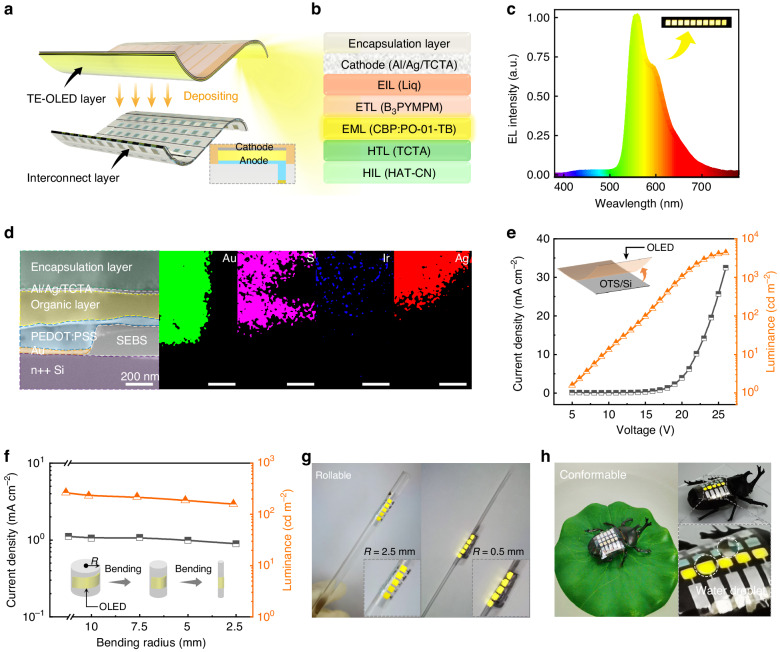
**Optical and electrical properties of conformable OLEDs**. **a** Schematic deposition of a TE-OLED on an interconnect layer. **b** Schematic diagram of the detailed description of each component and function of the TE-OLED. **c** Electroluminescence spectrum of OLED and a digital photograph of luminescence (inset). **d** Focused ion beam SEM image of a cross-section of TE-OLED with interconnect layers. Energy Dispersive Spectrometer (EDS) layered elemental mapping: Au (green), S (pink), Ir (blue), and Ag (red); showing the elemental distributions. **e** Current density-voltage-luminance characteristic curves for TE-OLED. **f** Current density and luminance at 16 V at different bending radii. **g**, **h** Photographs of the TE-OLED device adhering to glass sticks and beetles

TE-OLED has a high aperture ratio^43^. To ensure intimate contact between the TE-OLED and the interconnect layer, we directly deposited active layers onto the interconnect layer, then stripped the entire device from the OTS-modified silicon wafer with the aid of the NOA63 encapsulation layer mentioned earlier. Figure 3a, b shows the structure diagram and each component of the TE-OLED fabricated on the interconnect layer, respectively. In the fabrication of the interconnect layer mentioned earlier, the defined PEDOT:PSS micropattern acts not only as a filling hole but also as an OLED anode. Figure S9 shows a more detailed fabrication scheme for the PEDOT:PSS filling hole. Our modified PEDOT:PSS anode doubled the conductivity of 2392 S cm^−1^ compared with the pristine PEDOT:PSS (Fig. S10). To obtain a highly transparent and conductive cathode, Al/Ag/tris(4-carbazoyl-9-ylphenyl)amine (TCTA) was deposited on Liq film. Al (1 nm) as a seed layer to improve the continuity and conductivity of Ag film (Fig. S11a, b)^44^, and TCTA was introduced as a coupling layer to increase the transmittance of the whole cathode (Fig. S12)^45^. The transparent cathode obtained has transmittance up to 60% at 560 nm and square resistance as low as 4.1 Ω sq^−1^ (Figs. S12 and S13).

Energy-level matching between the functional layers of TE-OLEDs is the key to obtaining highly efficient devices. Figure S14 shows the TE-OLED interfacial energy band alignment. The host material for 4,4′-Bis(*N*-carbazolyl)-1,1′-biphenyl (CBP) is a low-polarity linear host material widely used as a control host for Iridium(III) bis(4-(4-tert-butylphenyl)thieno[3,2-c]-pyridinato-*N*,C2’)acetylacetonate (PO-01-TB) due to its similar linear conformation resembling a horizontal arrangement^46^. The CBP host has a higher triplet energy level *E*_T_ = 2.6 eV, which is greater than that of the orange PO-01-TB guest (2.2 eV) and facilitates efficient energy transfer and exciton blocking.

Figure 3c shows the normalized electroluminescence spectrum of the TE-OLED (at 1000 cd m^−2^). The device’s CIE coordinates (0.49, 0.50) remain stable under different bending radii (*R* = 10 − 2.5 mm), as shown in Fig. S15. This result is attributed to the NOA63 encapsulation, which protects the device from moisture and oxygen and increases device stability. To provide a clearer visualization of the device structure, an analysis using a focused ion beam-scanning electron microscope was conducted to inspect the device structure. It can be observed that the layers of the section are tightly interconnected and no delamination phenomenon occurs (Fig. 3d). Moreover, the thickness of each component layer is also apparent, and elemental Au, S, Ir, Ag, O, and N were detected by energy dispersive X-ray spectroscopy (Figs. 3d and S16).

The TE-OLED based on orange phosphorescent PO-01-TB fabricated on the interconnect layer exhibits remarkable electroluminescence. The maximum luminance of the device is 4296 cd m^−2^, the current efficiency is 40.48 cd A^−^^1^, the power efficiency is 11.56 lm W^−^^1^, and the external quantum efficiency is 12.96% (Figs. 3e and S17). To evaluate the TE-OLED as a component in flexible electronics, the electroluminescent characteristics were measured for different bending radii. Figures 3f and S18 show the current efficiency and external quantum efficiency for devices with flat surfaces and different bending radii. Furthermore, we investigated how device bending cycles affect the current density, brightness, current efficiency, and external quantum efficiency at 14 V to verify its mechanical and operational stability (see Fig. S19). Even when bent to a radius as small as 2.5 mm, the device maintains 80% of its initial current density. The slight decrease in luminance may be due to degradation of the interface layer, such as vacuum-evaporated dipyrazino[2,3-f:2’,3’-h]quinoxaline-2,3,6,7,10,11-hexacarbonitrile (HATCN), or altered interface contact caused by mechanical deformation. To visually demonstrate the mechanical flexibility of our TE-OLED device, we fixed it to various shapes, including two-dimensional curved glass tubes and 3D curved beetle surfaces. Figure 3g shows that the device operates normally even when wrapped around a glass tube with a radius of only 0.5 mm, which confirms the mechanical robustness of the device. Moreover, the device also demonstrates conformal capabilities for 3D surfaces. Figure 3h shows that the device can be fitted to the back of the beetle without folds and lights up normally when fixed to a 3D curved surface. The device encapsulated with transparent NOA63 not only retains its inherent flexibility but also becomes temporarily waterproof (Fig. S20). Therefore, if the device comes in contact with water, it continues to function normally, as displayed in the right panel of Fig. 3h. These results demonstrate that the conformable TE-OLED integrated with an interconnect layer has excellent photoelectricity properties, mechanical flexibility, and operational stability.

### All-photolithographic OTFTs

**Fig. 4:**
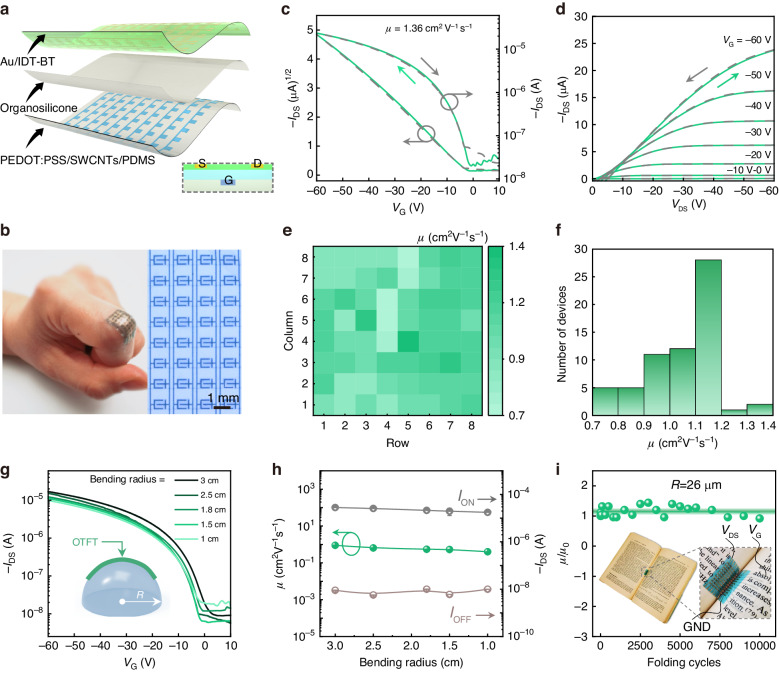
**Stability and uniformity of all-photolithographic OTFT arrays**. **a** Schematics of all-photolithographic BGTC OTFT array. **b** Photograph of the OTFT array conformable to finger joints and the transmission optical microscopy image of the OTFT array. **c**, **d** Typical transfer and output characteristics of the transistor array. **e**, **f** Color map and histogram distribution of field-effect mobility (8 × 8 devices). **g** Transfer characteristics at different bending radii. **h** Mobility, *I*_ON_, and *I*_OFF_ as functions of different bending radii. **i** Mobility changes after 10,000 folding cycles (*R* ≈ 26 μm)

The OTFT is an indispensable component of AMOLED displays because it provides a continuous and stable current for the OLEDs. Figure 4a shows structural diagrams of an all-photolithographic OTFT array, featuring a bottom-gate, top-contact configuration with indacenodithiophene-benzothiadiazole (IDT-BT) as the active layer. This configuration increases the charge injection area and exposes the source-drain electrode, ensuring a good connection with the OLED anode through the interconnect layer^37^. Moreover, the source, drain, and gate electrodes are all patterned by photolithography, facilitating the realization of various fine and complex patterns. Figure S21 provides a more detailed patterning scheme of the source, drain, and gate electrodes. Figure 4b shows an all-photolithographic OTFT array, which seamlessly adheres to a human finger joint and can move conformally, demonstrating its potential for applications in comfortable and wearable electronics.

We further evaluated the field-effect performance of an all-photolithographic OTFT array. Figure 4c, d shows its typical transfer and output characteristics. The channel width *W* = 1000 μm and the length *L* = 200 μm. The all-photolithographic OTFT demonstrates distinct *p*-type characteristics with clearly defined linear and saturation regions. More importantly, our all-photolithographic OTFT demonstrates excellent operating stability, with invisible hystereses in both transfer and output curves (Fig. 4c, d). In addition, the device exhibits a remarkable field-effect performance with a mobility *μ* as large as 1.36 cm^2^ V^−1^ s^−^^1^, a near-zero threshold voltage *V*_T_ as low as 0.2 V, and a current switching ratio *I*_on_/*I*_off_ > 10^5^. These characteristics are essential for achieving a skin-like display with a fast refresh rate, low power consumption, and controllable switching.

AMOLED displays require separate addressing of multiple pixels. OLEDs are driven by OTFTs, and their luminance uniformity depends heavily on the OTFT uniformity^39^. Moreover, given the mature development of skin-like AMOLED in practical applications, device consistency is a crucial factor. We measured 8 × 8 device arrays to confirm the good uniformity of our all-photolithography OTFT array. Figure 4e, f shows the spatial distribution and the statistical result for the corresponding field-effect performance. The average *μ* is 1.04 cm^2^ V^−1^ s^−^^1^ with a standard deviation of 0.14 cm^2^ V^−^^1^ s^−^^1^ and an average *V*_T_ of 1.69 V with a standard deviation of 1.15 V (Fig. S22). About 67% of the transistors have *μ* > 1 cm^2^ V^−^^1^ s^−^^1^, which is superior to amorphous silicon all-photolithographic OTFTs and approaches the level required for practical application. These results confirm that the proposed device array has good uniformity and provides a solid foundation for the subsequent fabrication of consistent skin-like AMOLED displays.

Mechanical flexibility is an essential guarantee for skin electronic products^12,13^. To investigate the mechanical flexibility of all-photolithographic OTFTs, we adhered arrays of devices to plastic hemispheres of different radii and measured the resulting field-effect properties. Figure 4g shows the corresponding transfer curves and bending diagram. Each device works well on plastic hemispheres with different radii. For a minimum bending radius *R* = 1 cm, the device retains the typical *p*-type field-effect performance. As the bending radius decreases from 3 cm to 1 cm, *μ,* and *I*_on_/*I*_off_ change slightly. The degradation of field-effect performance is due to interfacial strain caused by bending the semiconductor. Fixing the device array to plastic hemispheres introduces primarily tensile strain, which increases the intermolecular distance in the organic semiconductor. Fortunately, the mobility decreases negligibly under compressive strain. Figure 4i shows the correlation of *µ*/*µ*_0_ with a bending radius of 26 μm folded 10,000 times, where *µ*_0_ is the measured mobility in the flat state. Under the compressive strain caused by inward bending, *µ* remains essentially stable as the number of folds increases, and any change is reversible. More attractively, after 10,000 folding cycles, the mobility remains 92% of the initial mobility value. These extreme device configurations do not significantly alter the electrical properties, demonstrating that the proposed all-photolithographic OTFT possesses good mechanical flexibility and robustness.

### All-organic AMOLEDs

**Fig. 5:**
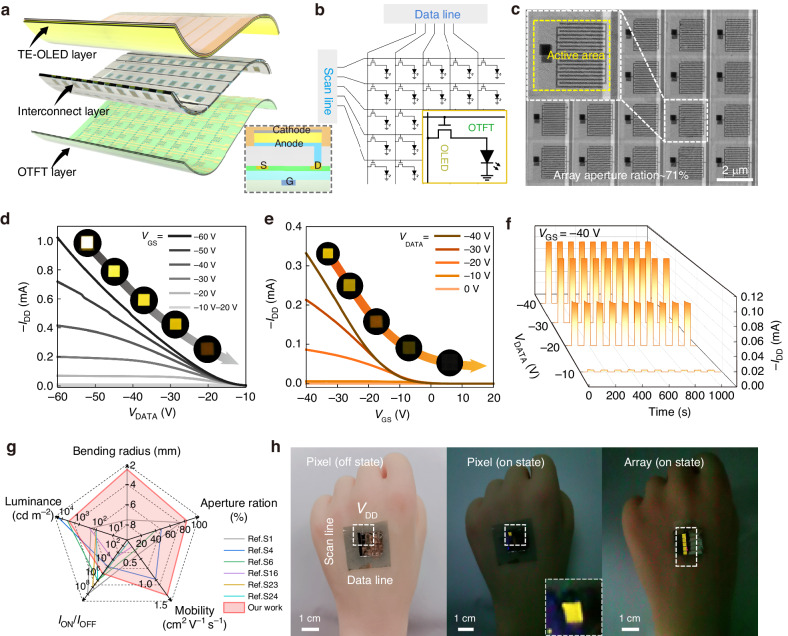
**Vertically stacked skin-like AMOLEDs**. **a** Schematics of vertically stacked AMOLED. **b** Schematic diagram of the AMOLED array control circuit. **c** AMOLED array optical microscope images. **d**, **e** The specific electrical performance of OLEDs controlled by driving transistors. Digital photographs of adjusting OLED luminance by changing *V*_GS_ and *V*_DATA_. **f** Plotting of pixel switching properties is controlled using different *V*_DATA_ repeatedly. **g** Device performance comparison of our device with reported all-organic flexible AMOLED displays. **h** Vertically stacked AMOLED arrays conformally fit on the back of an artificial hand. Digital photos with OTFT control of single and multiple pixels off and on

To achieve all-organic skin-like AMOLEDs, we stacked pre-prepared TE-OLEDs and all-photolithographic OTFTs tightly and vertically using a viscoelastic interconnect layer. Such a “discrete preparation-multilayer lamination” strategy avoids various adverse effects introduced during the preparation of each functional layer and renders the fragile organic materials compatible with modern microelectronics technologies such as photolithography and etching^37^. In addition, given that each layer is composed mainly of soft organic materials, the display has excellent flexibility and conformability. More strikingly, the vertically stacked structure avoids obstruction due to nonluminous areas (OTFT and wiring), maximizes the luminous TE-OLED area in each pixel, and dramatically improves the aperture ratio of the AMOLED display^7,10,19,33^. Another advantage of this structure is that the OTFT size need not be drastically reduced to obtain a high aperture ratio, which facilitates the design of complex driver circuits. Figure 5a, b shows structural diagrams and a circuit schematic, respectively, of an AMOLED display^18^. A single OTFT drives a single OLED pixel to achieve OTFT-OLED (1T-1D). The scan line denotes the OTFT gate voltage *V*_GS_, and the data line denotes the bias voltage *V*_DATA_ of 1T-1D. By scanning *V*_GS_ or *V*_DATA_ of the OTFT, the pixel current *I*_DD_ flowing from the drain electrode to the TE-OLED anode can be accurately controlled, thereby allowing the modulation of the TE-OLED luminance so that it can be turned on and off^12,13,39^.

Figure 5c presents optical micrographs of a vertically stacked AMOLED array. The area of the TE-OLED is 2000 μm × 2000 μm, and the all-photolithographic OTFT driver has dimensions of 20,400 μm/50 μm in a multi-finger design. The calculated aperture ratio of the array is close to 71%, which exceeds the aperture ratio of all previously reported all-organic AMOLED arrays. Figure 5d, e shows the electrical performance of TE-OLEDs controlled by driving transistors. Unlike the transfer characteristics, the minimum threshold voltage (*V*_T_OLED_) of the TE-OLED must be taken into account in the output characteristics due to its diode-like behavior. Consequently, *V*_DATA_ greater than 7 V is essential to activate the circuit. When *V*_GS_ = −30 V and *V*_DATA_ = −2.5 V, *V*_DATA_ < *V*_T_OLED_ of the OLED (7 V), so the OLED is off. The all-photolithography OTFT device has a milliamp-amplitude current, which satisfies the requirements for the TE-OLED operating current. The OLED electroluminescence is well-modulated by tuning *V*_DATA_ and *V*_GS_. Figure 5d shows the OLED emitting maximum luminance at different *V*_GS_, increasing *V*_DATA_ leads to a gradual decrease in current, from fully on to a gradual reduction in luminance (shown in the illustration). Figure 5e shows the process of OLED device luminance from half on to gradually decreasing and then to closing as *V*_GS_ increases and the current gradually decreases under different *V*_DATA_ (see the illustration). Figure 5f shows *I*-time testing on the device to study its stability and response speed during long-term operation. With *V*_GS_ = −40 V and repeated *V*_DATA_ pulses of −10 V, −20 V, −30 V, and −40 V, the OLED switches between stable *I*_ON_ and *I*_OFF_ states. For 1000 s, the electrical performance of the AMOLED remains stable over ten continuous opening and closing cycles.

Figure 5g shows the statistical results of all-organic flexible AMOLED displays reported in recent years. Table S1 presents a detailed performance checklist. The proposed AMOLED display offers outstanding advantages in terms of bending radius and aperture ratio, mainly due to its all-organic composition and vertical integration. High mechanical flexibility ensures the normal operation of the display under large deformation, and the high aperture ratio increases the luminance of the display, thus producing a high-quality display. Using the same strategy to obtain AMOLED (2T1C) should significantly improve the aperture ratio.

To further demonstrate that the proposed AMOLED display functions properly under deformation, we attached the display to a silicone prosthetic hand, as shown in Fig. 5h. Note that the display fitted the prosthetic hand as closely as the skin. The pixel was switched on and off independently by using all-photolithographic OTFT regulation. The AMOLED array simultaneously controlled multiple pixels to display a pattern of “1”. These results confirm that the proposed strategy enables the fabrication of skin-like AMOLED displays with high aperture ratios that have significant potential for the development of next-generation wearable electronics such as skin-like displays.

## Discussion

We propose herein a simple and general integrating strategy for vertically stacked skin-like active-matrix displays. The interconnect layers are prepared using a nondestructive photolithography approach, allowing us to achieve high-precision, large-area via holes and filling holes to accurately satisfy the requirements of electrical isolation and electrical connection between functional layers. By vertically integrating TE-OLED and all-photolithography OTFTs with interconnect layers, we fabricate ultralight, ultra-conformal, and ultrahigh-aperture-ratio skin-like displays. This integration strategy solves many challenges faced by vertically stacked skin-like AMOLEDs, including mutual dissolution, mechanical compatibility, and interconnectivity between functional layers. The results show that the key metrics are significantly better than those reported earlier for all-organic AMOLEDs. This study thus provides a solution for vertically stacked skin-like active-matrix displays composed of all-organic materials and demonstrates the potential for commercial development of wearable electronics and optoelectronics^47–49^.

## Materials and methods

### Materials

All materials are purchased commercially and used based on the received materials. SEBS H1052 was purchased from Asahi Kasei Corporation. Poly (3,4-methylenedioxythiophene):poly-(styrene sulfonate) (PEDOT:PSS, PH1000) was acquired from Heraeus. Bis(trimethylsilyl)amine, hexamethyldisilazane (HMDS, 95%), and PVA (Mw ≈ 205,000 g mol^−1^) were acquired from Sigma-Aldrich. Polymer semiconductors materials, including DPPT-TT and IDT-BT (99%) were acquired from Derthon Optoelectronic Materials Co. Ltd. Organosilicone (DC 1-2577) was acquired from Dow Corning and was dissolved in OS-20 (Dow Corning Co.). Surfactants (Capstone FS-30) and ethylene glycol (EG) were provided by DuPont and Sigma-Aldrich, respectively. The single wall carbon nanotubes (SWCNTs) aqueous dispersion (0.2 wt%) was provided by Chengdu Organic Chemicals Co. Ltd, Chinese Academy of Sciences. Pentafluorobenzenethiol (PFBT, 95%) and octadecyltrichlorosilane (OTS, 90%) were commercially available and provided by TCI Co. Ltd and Acros Corporation, respectively. Sylgard-184 is a commonly used PDMS material that was provided by Dow Chemical Company. NOA63 was provided by Norland Corporation. All small-molecule organic semiconductors used to prepare OLED devices were purchased from Xi’an Yuri Solar Co., Ltd.

### Fabrication of conformable interconnect layer

Silicon wafers were hydroxylated with an O_2_ plasma treatment (100 W, 3 min). Then immersed in OTS:n-heptane (1:1000) solution for 1 h. Following hydrophobic treatment of the silicon wafer, SEBS solution (30 mg ml^−1^ toluene solution) was spin-coated on the silicon wafers (4000 rpm, 40 s), and cured for 1 h at 100 °C in a hotplate. Immediately thereafter, the PVA solution and DPPT-TT solution were spin-coated (6000 rpm, 30 s) onto the substrate, and cured on a hot plate (5 min, 60 °C). Sequentially, photoresist (AZ5214E) was spin-coated on smooth DPPT-TT, and cured in a hotplate (3 min, 100 °C). Following exposure, development, and fixation of the samples by traditional photolithography, the samples were transferred to an oxygen plasma for etching (100 W, 10 min) to remove the areas of SEBS/PVA/DPPT-TT that were not covered by the photoresist, which resulted in the successful realization of a patterned via hole layer. To ensure that the stripping process would not cause damage to the devices, the samples were treated with hydrophobic modification again. Following the 30 nm gold pre-fill hole layer was fabricated by the traditional photolithography lift-off process. The PEDOT:PSS post-fill hole layer (mixed 2% FS-30) was patterned based on our non-destructive photolithography approach, the same as for the interconnect layer. Of special note, to protect the PEDOT:PSS films from damage by water and other solvents during photolithography, before photolithography patterning immersed them in concentrated nitric acid for 3 min and subsequently rinsed them with distilled water. Finally, the fabricated interconnect layer was separated from the OTS-modified silicon wafer using a simple dry peeling and then transferred onto any material surface.

### Fabrication of conformable OLED array on interconnect layer

After filling the interconnect layer with PEDOT:PSS, the TE-OLED is fabricated on the interconnect layer immediately, aiming to strengthen the stable connection between the functional layers. It is worth noting that the PEDOT:PSS here is used not only as a hole filler but also as an anode for TE-OLED. To protect the PEDOT:PSS films from water and other solvents during photolithography, they were immersed in concentrated nitric acid for 3 min prior to photolithography patterning and subsequently rinsed with distilled water. Subsequently, the PDOT:PSS anode was patterned using photolithography and removed excess material with oxygen plasma (100 W, 5 min). It is worth noting that the photolithography process of PEDOT:PSS is the same as that of the interconnect layer.

Next, the devices were deposited layer by layer under vacuum conditions below 5.0 × 10^−4^ Pa. The structure of the TE-OLED device consists of 30-nm-thickness PEDOT:PSS/10-nm-thickness HATCN/25-nm-thickness TCTA/20-nm-thickness emissive layer (EML)/35-nm-thickness B_3_PYMPM/1-nm-thickness Liq/1-nm-thickness Al/15-nm-thickness Ag/40-nm-thickness TCTA. Wherein, the EML was a thin film doped with 8 wt% PO-01-TB in the CBP host. HATCN and TCTA were used as the hole-injection layer (HIL) and hole-transportation layer (HTL), respectively. B_3_PYMPM and Liq were used as the electron-transportation layer (ETL) and electron-injection layer (EIL), respectively. PEDOT:PSS and Al/Ag/TCTA were used as anode and cathode, respectively. After deposition layer by layer, to prevent water vapor and oxygen from entering the device, we quickly encapsulated the OLED device with NOA63. The NOA63 utilized a step-by-step spin-coated strategy, first (500 rpm for 10 s) and then (4000 rpm for 30 s). Curing was then achieved by exposure to UV light at 365 nm wavelength for 40 s. After curing, the NOA63/OLED/interconnect layer was slowly peeled off together with tweezers and then flipped over to form the TE-OLED with interconnect layer.

### Fabrication of conformable BGTC-OTFT arrays

(i) Micropatterned source-drain electrodes (30 nm gold) were fabricated on a silicon wafer modified with OTS by the lift-off photolithography process. After removing the photoresist, the electrode was immersed in a PFBT solution (PFBT:toluene = 1:1000 by volume) at room temperature and atmospheric conditions for 2 min. (ii) The polymer semiconductor IDT-BT (5 mg ml^−1^ chloroform solution) was spin-coated on the source-drain electrodes (1500 rpm, 60 s), and then transferred to a vacuum drying oven and annealed in an N_2_ atmosphere (100 °C, 30 min). The elastic dielectric DC 1-2577 solution (DC 1-2577:OS-20 = 1:5 by volume) was spin-coated (6000 rpm, 60 s) on the semiconducting layer, and cured in a drying oven (60 °C, 30 min). (iii) The gate electrodes (PEDOT:PSS/SWCNTs hybrid electrodes) were fabricated by the traditional photolithography process. PEDOT:PSS/SWCNTs hybrid electrodes were fabricated on a clean OTS/Si substrate. Then, PDMS (crosslinking ratio of 10:1) was spin-coated (1000 rpm, 30 s) on the gate electrode, and cured in a drying oven (70 °C, 30 min). (iv) PDMS-embedded gate electrodes were slowly peeled off from the OTS-modified silicon wafer and flipped over. Afterward, the PEDOT:PSS/SWCNTs gate electrodes were aligned with source-drain electrodes with the semiconductor and dielectric layer under the microscope. After 10 min of heat treatment, they formed close contact with each other. Finally, the whole device was stripped (PDMS support layer/gate electrodes/dielectric layer/semiconductor layer/source-drain electrodes) from the OTS-modified silicon wafer to obtain conformal BGTC-OTFT arrays.

### Fabrication of the conformable AMOLED arrays

The TE-OLED with interconnect layer was laminated on the all-photolithographic BGTC-OTFT under the microscope to form skin-like active-matrix displays. The anode of the TE-OLED was connected to the drain electrode of the all-photolithographic BGTC-OTFT through the internal interconnect layer. Finally, the whole device (OLED/interconnect layer/OTFT) was peeled off from the supporting layer to form the vertically stacked skin-like AMOLED.

### Characterizations

Optical image investigations were obtained with a Keyence VHX-5000 3D optical microscope (Keyence, Japan). Scanning electron microscopy (SEM) images were taken with a Philips XL30 instrument. AFM was taken on a Dimension Icon instrument using the NanoScopeV9 controller (Bruker, Inc.). The capacitance characteristic curves were acquired by the IM 3590 chemical impedance analyzer. Fourier transform infrared (FTIR) spectroscopy was recorded using Nicolet 6700 by Thermo. Raman spectra were recorded on a HORIBA LabRam Raman spectrometer. The excitation source was chosen from a He-Cd laser with a 488 nm wavelength. A UV-vis-NIR spectrophotometer with absorption mode (Hitachi, UH4150) was used to record the absorption spectra. A spectrophotometer (PR655, Photo Research) in combination with a source meter (Keithley 2400) was used to measure the basic optoelectronic characteristics of the top-emitting OLEDs. All the tests were carried out under atmospheric conditions at room temperature.

The electrical characteristics of OTFT device parameters were calculated with the standard equation in the saturation regime. The standard equation is

$$\mu =\frac{2L}{W{C}_{i}}{\left(\frac{\root{\mathrm{\partial}}\of{{{I}_{{DS}}}}}{\partial {V}_{G}}\right)}^{2}$$

Where *W* and *L* are channel width and channel length, respectively. The electrodes are interdigitated electrodes, and the width-to-length ratio of the channel is 5/1 (*L* = 200 µm, *W* = 1000 µm). *C*_*i*_ is the unit area capacitance of DC 1-2577, which is 2.34 nF cm^−2^ at 1 Hz, *I*_DS_ is the drain current, and *V*_GS_ is the gate voltage.

The aperture ratio refers to the ratio between the area of light that passes through each sub-pixel after removing the wiring and transistor parts (usually hidden by the black matrix) and the total area of the sub-pixel. The standard equation is:

$${{\rm{Aperture}}\; {\rm{ratio}}}=\frac{a\times b}{{xy}}\times 100 \%$$

Where *x* and *y* are unit pixel area length and width, respectively. Where *a* and *b* are active area length and width, respectively (Fig. S23).

The experiments involving human subjects have been performed with the full, informed consent of the volunteers and were in compliance with IRB/Committee approval.

### Supplementary information

Supporting information for Vertically stacked skin-like active-matrix display with ultrahigh aperture

## Data Availability

Data is available from corresponding author upon reasonable requirements.
