# Supplementary material for: Vertically stacked skin-like active-matrix display with ultrahigh aperture ratio
Source: Light Sci Appl. 2024 Jul 26;13:177. doi: 10.1038/s41377-024-01524-z (PMC11282298; doi:10.1038/s41377-024-01524-z)
Supplement: Supplementary file 1 — Supporting information for Vertically stacked skin-like active-matrix display with ultrahigh aperture [file 41377_2024_1524_MOESM1_ESM.pdf]

Supplementary Information

Vertically stacked skin-like active-matrix display with ultrahigh aperture ratio

Juntong Li[1], Yanping Ni[1], Xiaoli Zhao[1]*, Bin Wang[1], Chuang Xue[1], Zetong Bi[1], Cong Zhang[1], Yongjun Dong[1]*, Yanhong Tong[1], Qingxin Tang[1]* and Yichun Liu[1]

Correspondence: Qingxin Tang (tangqx@nenu.edu.cn) or Xiaoli Zhao (zhaoxl326@nenu.edu.cn) or Yongjun Dong(dongyj512@nenu.edu.cn)

[1]Key Laboratory of UV-Emitting Materials and Technology of Ministry of Education, Northeast Normal University, Changchun 130024, China

Table S1. Comparison of this study with recently reported all-organic active-matrix display.

Structure	Aperture ratio (%)	Mechanical performance	Mobility (cm^2 V^{-1} s^{-1})	I_{ON}/I_{OFF}	Luminance (cd/m^2)	Ref.
Vertical	75	Flexible	0.4	10^8	200	1
Parallel	54	Rigid	0.6	10^7	-	2
Parallel	52	Flexible	0.2	10^7	-	3
Vertical	48	Flexible	0.75	10^5	16636	4
Vertical	44	Rigid	2.5	10^8	>150	5
Vertical	39	Stretchable	0.3	10^6	5000	6
Parallel	38	Rigid	0.7	10^6	100	7
Parallel	37	Flexible	0.25	10^3	50	8
Parallel	34	Flexible	1	10^7	-	9
Parallel	30	Flexible	-	10^8	-	10
Parallel	27	Rigid	2	10^5	400	11
Parallel	26.3	Flexible	0.31	10^7	100000	12
Parallel	23	Flexible	0.2	-	70000	13
Parallel	22	Flexible	0.23	10^6	64459	14
Parallel	-	Rigid	30.6	10^4~10^5	-	15
Vertical	-	Flexible	0.21	10^3	260	16
Parallel	-	Flexible	0.8	10^5	300	17
Parallel	-	Rigid	0.4	10^5	600	18
Parallel	-	Flexible	0.28	10^7	-	19
Parallel	-	Rigid	0.13	10^6	90	20
Parallel	-	Flexible	1	10^6	>1000	21
Vertical	-	Flexible	0.1	10^6	>100	22
Parallel	-	Flexible	0.05-0.1	10^6	30	23
Parallel	-	Flexible	0.04	10^3	>200	24
Vertical	83	Flexible	1.36	10^5	4000	

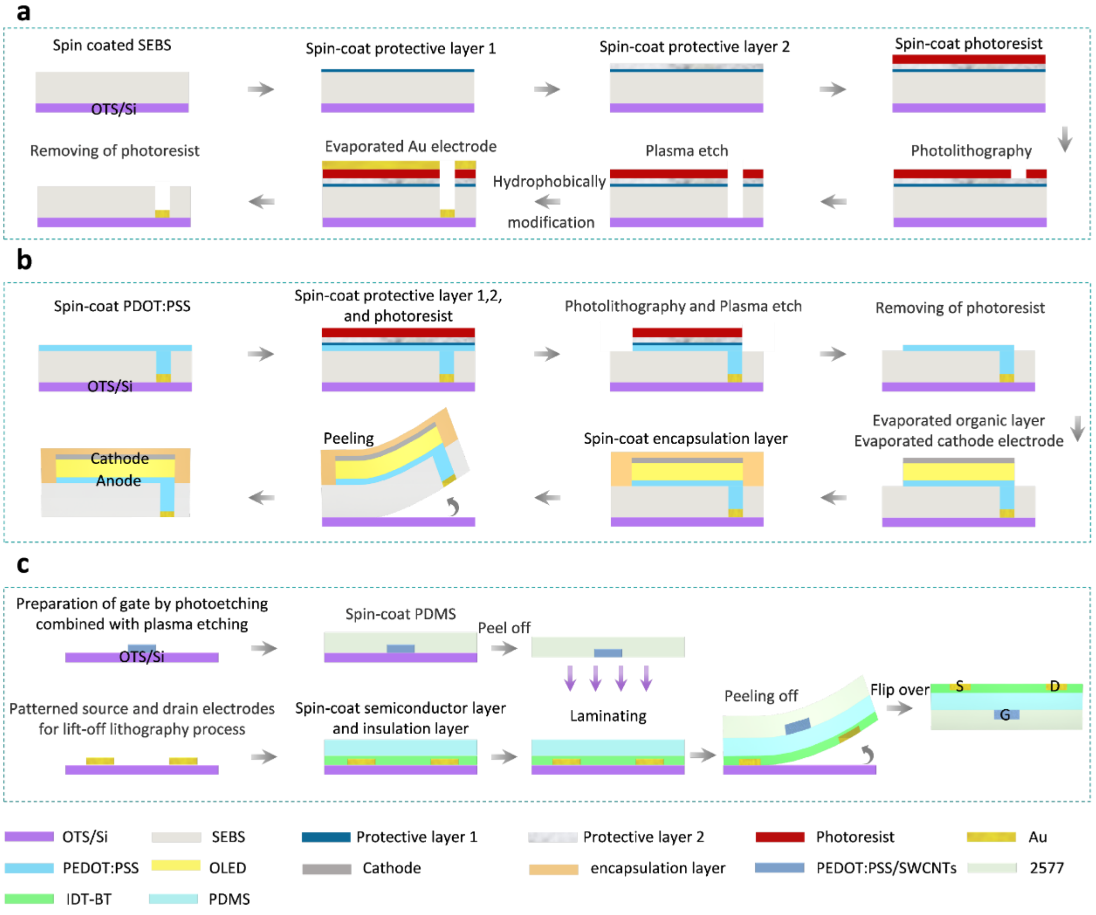

Fig. S1. Skin-like AMOLED array fabrication scheme. (a) Fabrication schematic diagram of conformable interconnection layer. (b) Fabrication schematic diagram of conformable OLED on the interconnection layer. (c) Fabrication schematic diagram of the conformable BGTC-OTFT. Fabrication schematic diagram of the conformable skin-like AMOLED is shown in the fig. 1a (I, II, III, and IV).

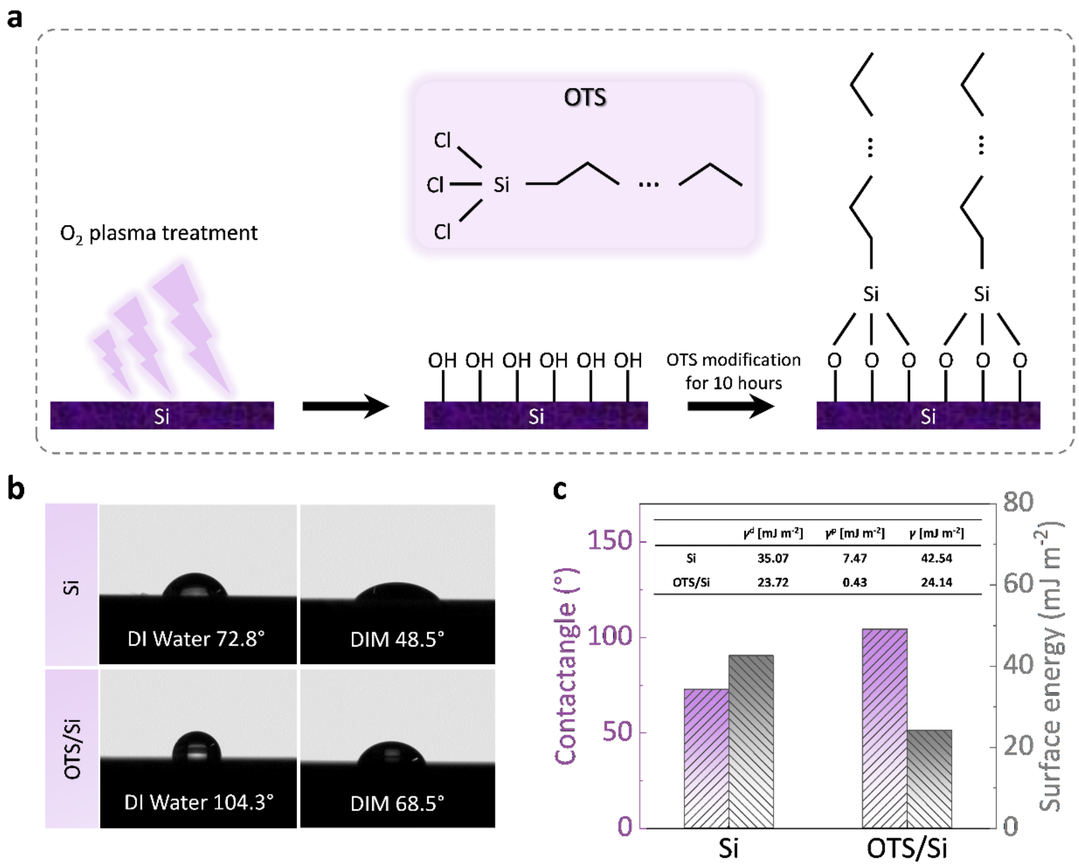

Fig. S2. (a) Schematic diagram of OTS modification mechanism. (b, c) Contact angle and surface energy corresponding to silicon wafers with and without OTS modification.

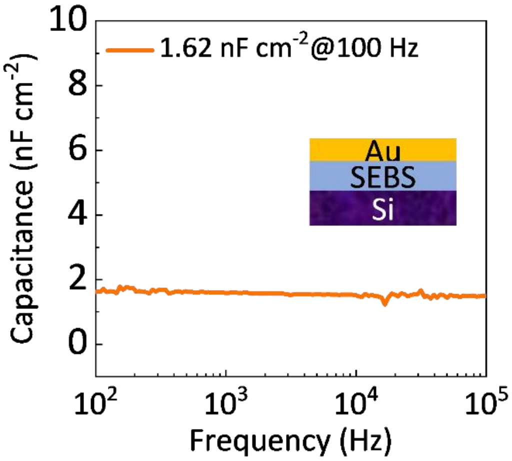

Fig. S3. Capacitance-frequency curves of the interconnect layer. The inset shows the schematic diagram of the test structure.

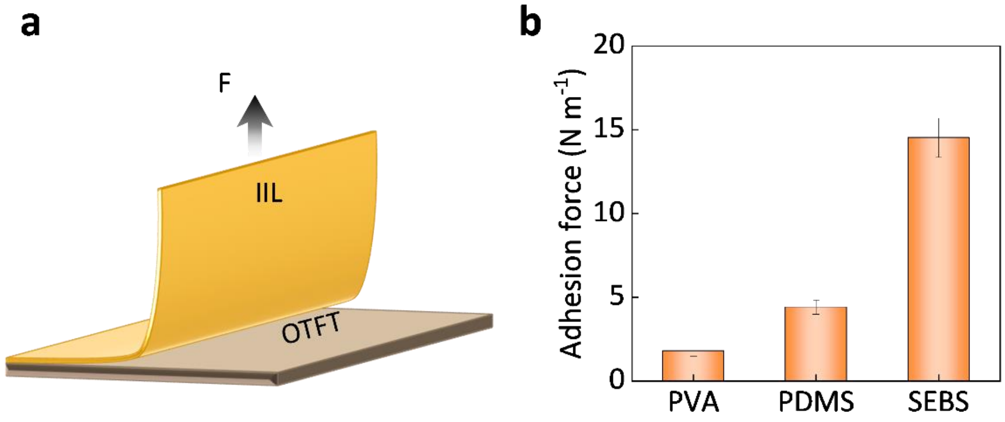

Fig. S4. (a) Schematic diagram of 90° peeling tests of interconnecting layers. (b) Adhesion force of PVA, PDMS, and SEBS films on OTFTs.

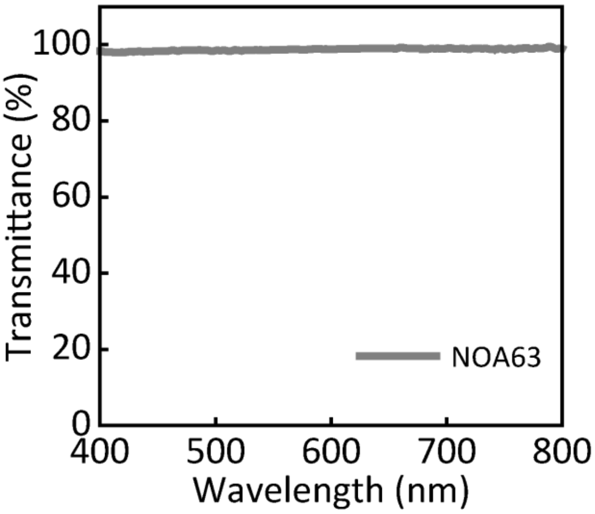

Fig. S5. Optical transmittance of the NOA63.

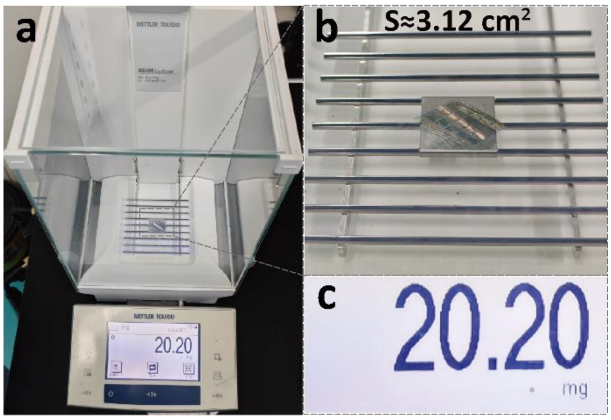

Fig. S6. (a) The picture of the weight measurement. (b) The 2.4 × 1.3 cm^2 AMOLED array was placed on the measuring table of an electronic balance. (c) The test weight of the whole device is 20.2 mg. Therefore, we can calculate that the mass of the device per unit area is 65 g m^{-2}.

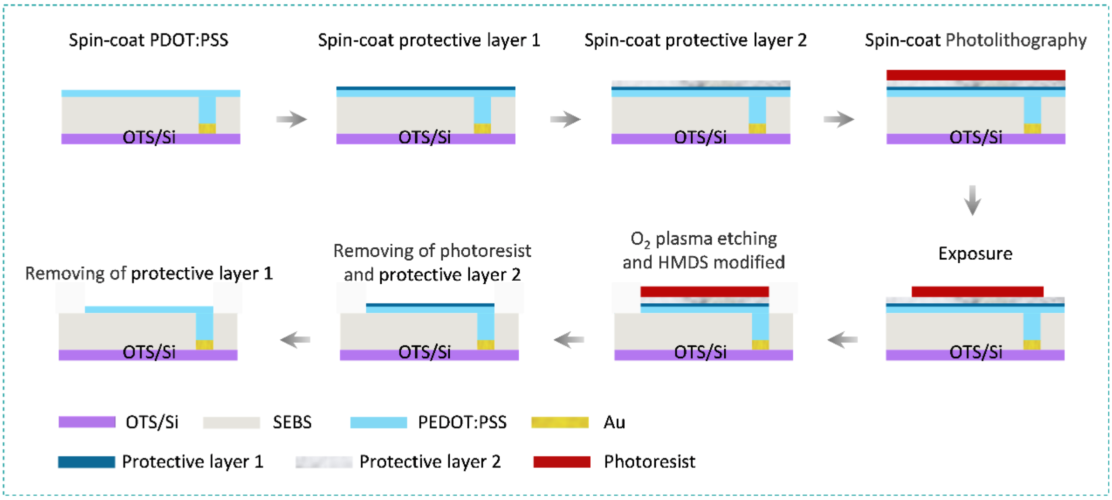

Fig. S7. Schematic diagram of the detailed preparation process of the PEDOT:PSS electrodes.

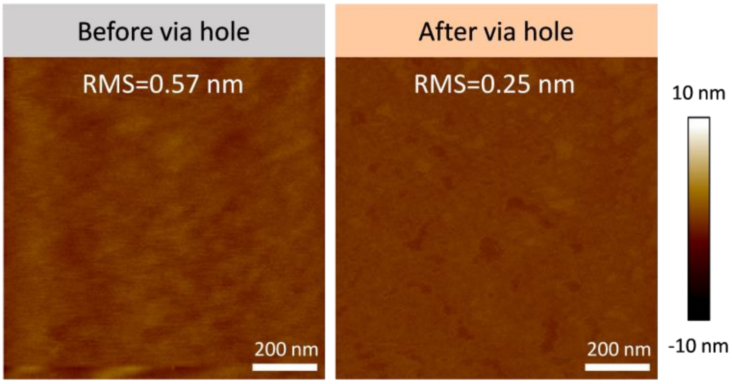

Fig. S8. AFM image of the SEBS before and after the via hole. Scale bar: 200 nm.

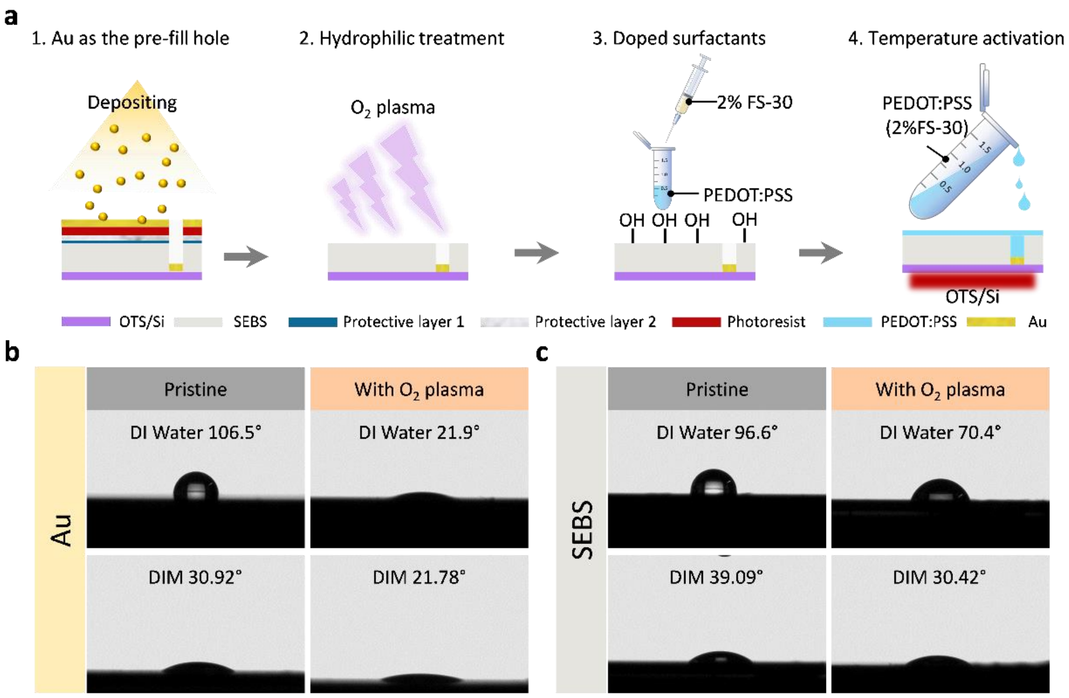

Fig. S9. (a) Schematic diagram of the main fabrication scheme for PEDOT:PSS filling holes. (b) Contact angle of pristine Au and Au with O_2 plasma treatment. (c) Contact angle of pristine SEBS and SEBS with O_2 plasma treatment.

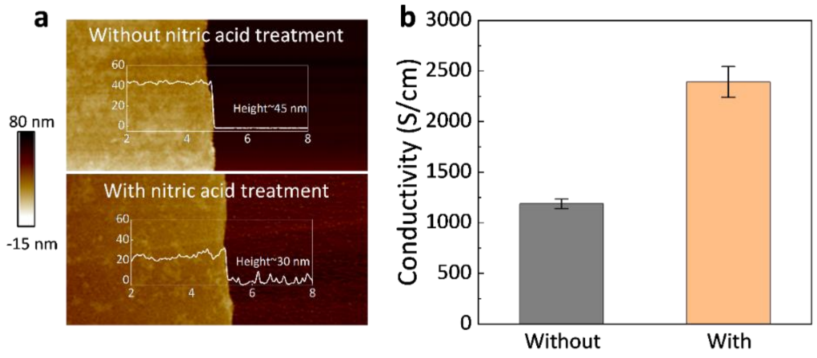

Fig. S10. (a) AFM images of anodes (PEDOT:PSS) without and with nitric acid treatment film with thicknesses. (b) Conductivity of without and with nitric acid treatment anodes (PEDOT:PSS).

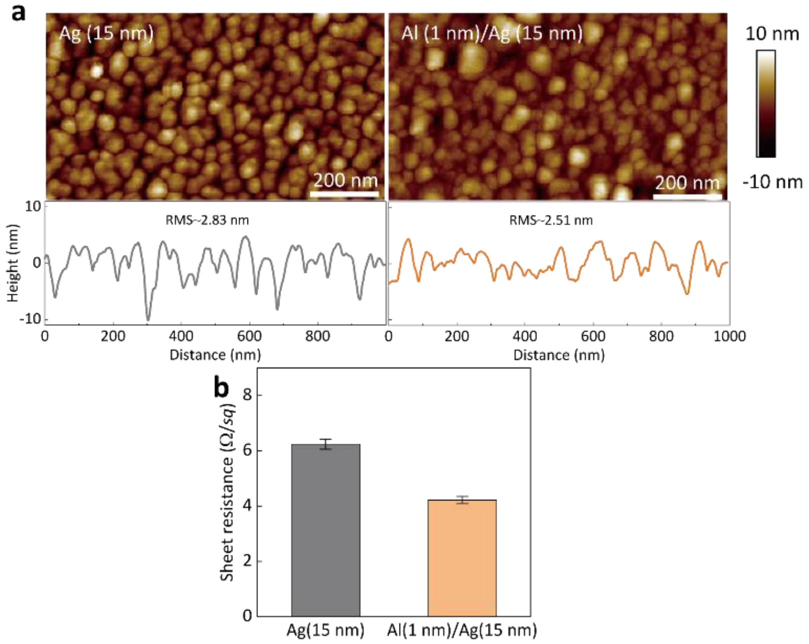

Fig. S11. (a) AFM images of pure Ag (15 nm) film and laminated Al (1 nm)/Ag (15 nm) film. (b) Sheet resistance of the Ag and Al/Ag cathode.

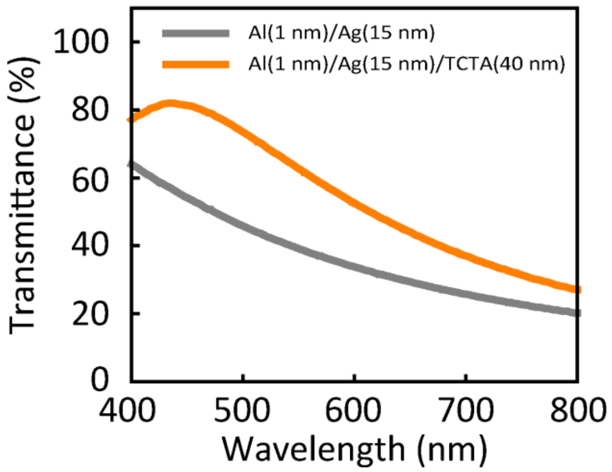

Fig. S12. Optical transmittance of cathodes without and with TCTA (40 nm) as coupling layer.

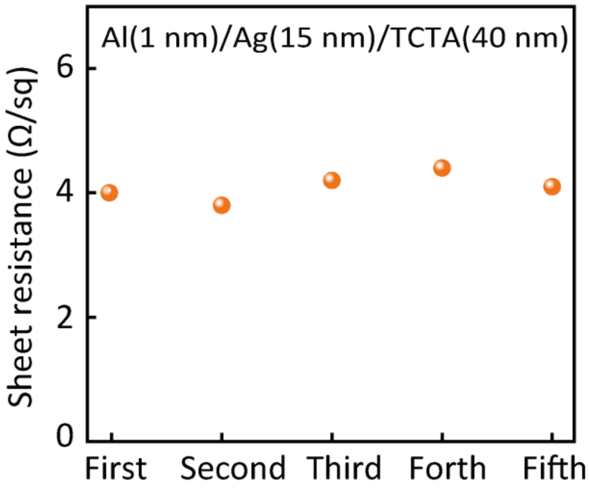

Fig. S13. Sheet resistance of Al/Ag/TCTA cathodes on five different substrates.

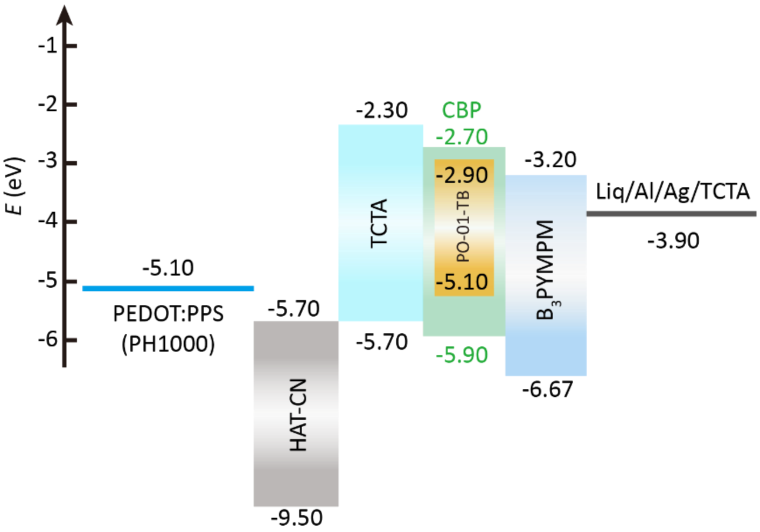

Fig. S14. Energy-level alignment diagram for the top-emitting OLED.

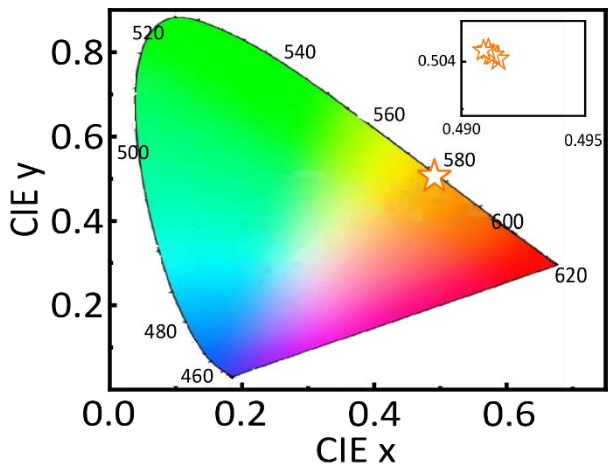

Fig. S15. CIE coordinates of top-emitting OLED under different bending radii (*R*=10 mm~2.5 mm).

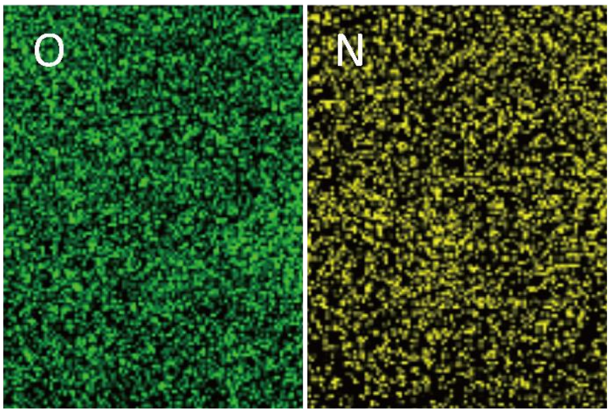

Fig. S16. The corresponding elemental spectra of O and N were detected by energy dispersive X-ray (EDX) spectroscopy.

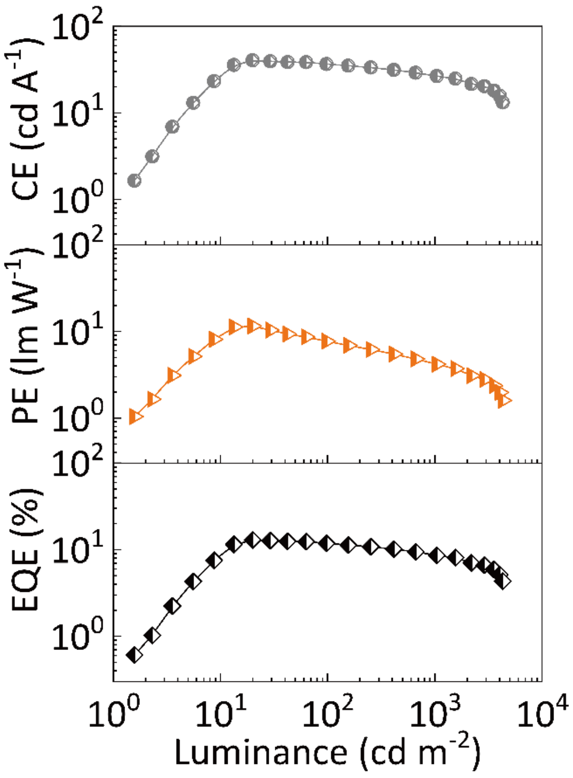

Fig. S17. Current efficiency, power efficiency, external quantum efficiency, and luminance characteristics of top-emitting OLED fabricated on the interconnect layer.

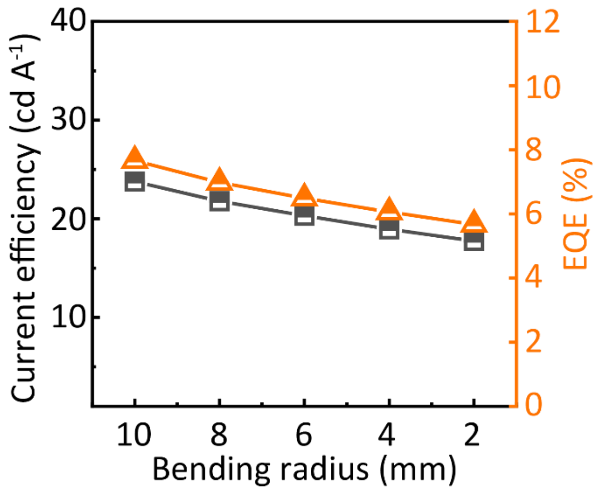

Fig. S18. Current efficiency and external quantum efficiency under different bending radii (R=10 mm~2.5 mm).

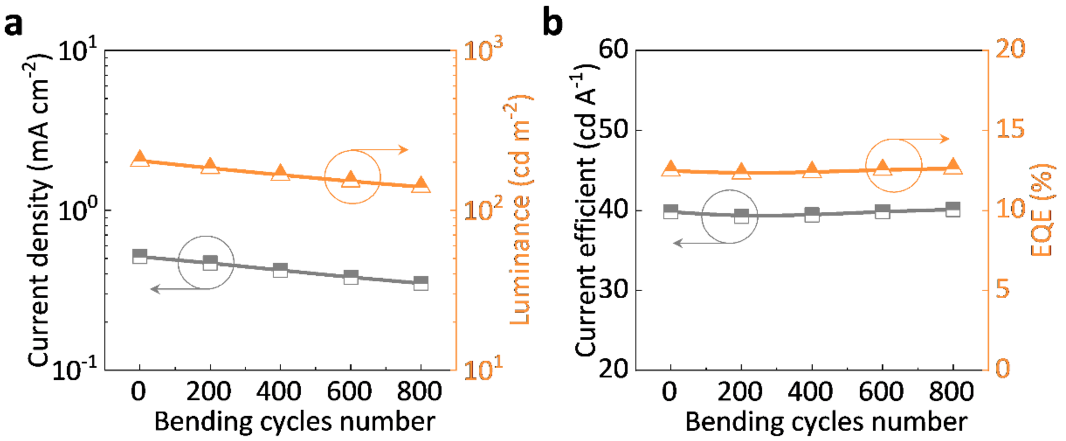

Fig. S19. Changes in current density, brightness, CE, and EQE of the device at 14 V for different bending cycles (at a bending radius of 5 mm).

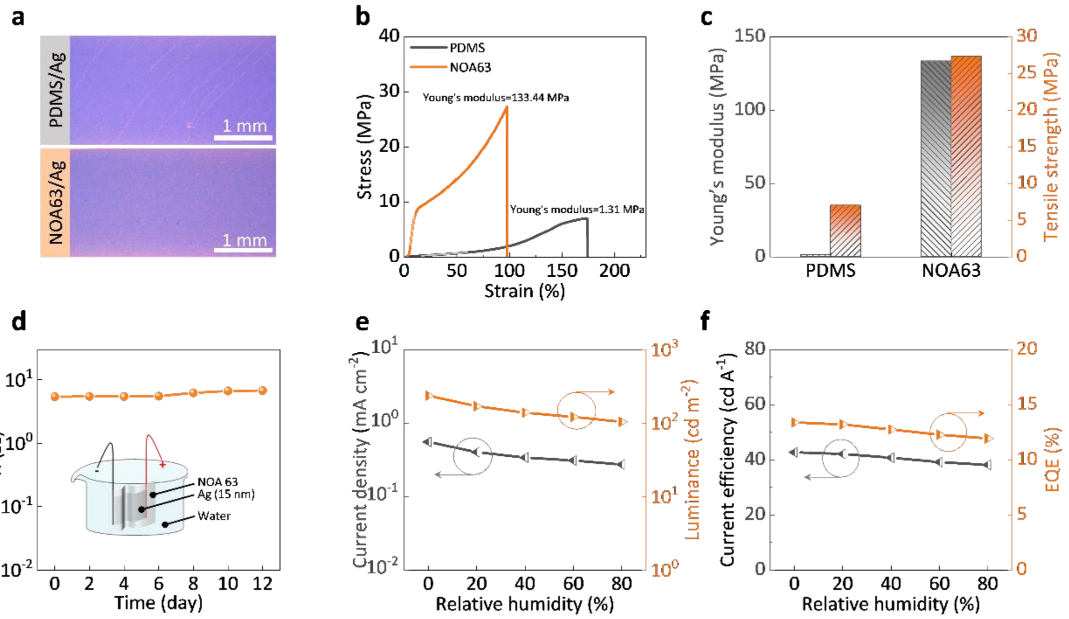

Fig. S20. (a) Optical microscope images. Scale bar: 1 mm. (b) Stress-strain curves of NOA63 and PDMS encapsulation layers. (c) Young's modulus and tensile strength of NOA63 and PDMS encapsulation layers. (d) Resistance changes with time of the NOA63 encapsulated Ag electrode. (e-f) OLED device performance characteristic curves under different RH conditions (20%, 40%, 60%, and 80%).

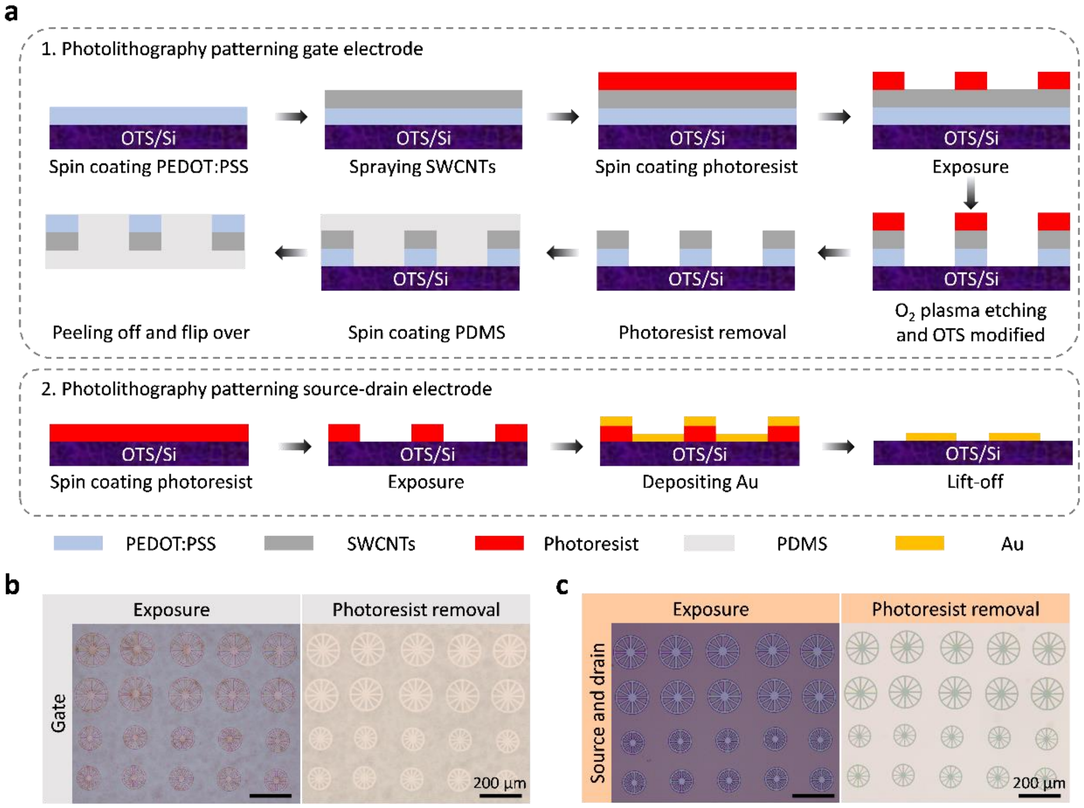

Fig. S21. (a) Schematic diagram of the detailed preparation process of the source/drain/gate electrodes. (b) Microscope images of fine patterning of gate electrodes. Scale bar: 200 μm. (c) Microscope images of fine patterning of source/drain electrodes. Scale bar: 200 μm.

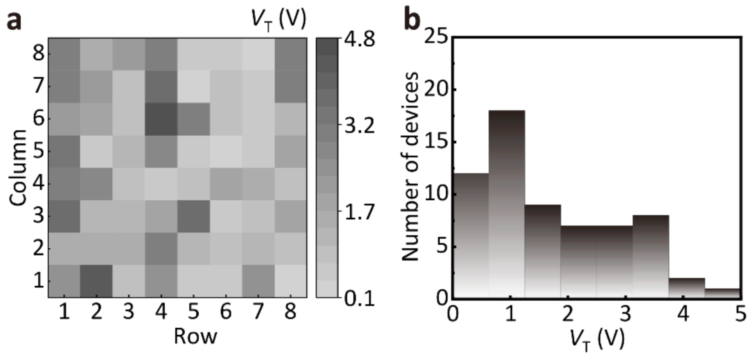

Fig. S22. Threshold voltage color maps of an all-photolithographic OTFT array with the device number of 8 × 8 devices.

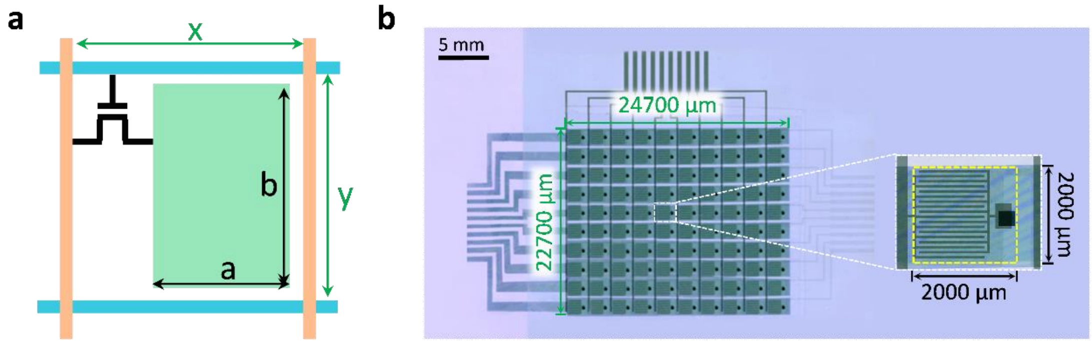

Fig. S23. (a) Schematic diagram of aperture ratio calculation. (b) 3D optical microscope images of the prepared vertically stacked AMOLED array with actual device size.

Supplementary References

1. Steudel, S. et al. Design and realization of a flexible QQVGA AMOLED display with organic TFTs. *Organic Electronics* **13**, 1729-1735 (2012).
2. Zhou, L. S. et al. Pentacene TFT driven AMOLED displays. *IEEE Electron Device Letters* **26**, 640-642 (2005).
3. Zhou, L. S. et al. All-organic active matrix flexible display. *Applied Physics Letters* **88**, 083502 (2006).
4. Kim, J. S. et al. Textile Display with AMOLED Using a Stacked-Pixel Structure on a Polyethylene Terephthalate Fabric Substrate. *Materials* **12**, 2000 (2019).
5. Okumoto, Y. et al. Top-emission AMOLED display driven by organic TFTs with semiconductor layer patterned by inkjet process. *Journal of the Society for Information Display* **20**, 575-580 (2012).
6. Sekitani, T. et al. Stretchable active-matrix organic light-emitting diode display using printable elastic conductors. *Nature Materials* **8**, 494-499 (2009).
7. Lee, S. et al. Development of High-Performance Organic Thin-Film Transistors for Large-Area Displays. *MRS Bulletin* **31**, 455-459 (2006).
8. Mizukami, M. et al. Flexible AMOLED panel driven by bottom-contact OTFTs. *IEEE Electron Device Letters* **27**, 249-251 (2006).
9. Han, S. H. et al. High Resolution OTFT-OLED on Plastic Substrate Using Self-organized Process. *SID Symposium Digest of Technical Papers* **38**, 1757-1760 (2007).
10. Yamamoto, T. et al. New Driving Scheme to Improve Hysteresis Characteristics of Organic Thin Film Transistor-Driven Active-Matrix Organic Light Emitting Diode Display. *Japanese Journal of Applied Physics* **50**, 024201 (2011).
11. Ohta, S. et al. Active Matrix Driving Organic Light-Emitting Diode Panel Using Organic Thin-Film Transistors. *Japanese Journal of Applied Physics* **44**, 3678-3681 (2005).

12. Ryu, G. S. et al. A printed OTFT-backplane for AMOLED display. *Organic Electronics* **14**, 1218-1224 (2013).
13. Yan, J. Y. et al. 3.1-inch Flexible Top-Emitting AMOLED on Plastic Substrate Driven by Organic Thin Film Transistors. *SID Symposium Digest of Technical Papers* **2**, 986-987 (2009).
14. Kim, J. S. et al. AMOLED panel driven by OTFTs on polyethylene fabric substrate. *Organic Electronics* **30**, 45-51 (2016).
15. Ji, D. Y. et al. Surface Polarity and Self-Structured Nanogrooves Collaboratively Oriented Molecular Packing for High Crystallinity toward Efficient Charge Transport. *Journal of the American Chemical Society* **139**, 2734-2740 (2017).
16. Zyung, T. et al. Flexible Organic LED and Organic Thin-Film Transistor. *Proceedings of the IEEE* **93**, 1265-1272 (2005).
17. Fujisaki, Y. et al. Flexible Active-Matrix Organic Light-Emitting Diode Display Using Air-Stable Organic Semiconductor of Dinaphtho[2, 3-b: 2', 3'-f]thieno[3, 2-b]-thiophene. *IEEE Transactions on Electron Devices* **59**, 3442-3449 (2012).
18. Tsukagoshi, K. et al. Organic light-emitting diode driven by organic thin film transistor on plastic substrates. *Journal of Applied Physics* **99**, 064506 (2006).
19. Mizukami, M. et al. A Solution-Processed Organic Thin-Film Transistor Backplane for Flexible Multiphoton Emission Organic Light-Emitting Diode Displays. *IEEE Electron Device Letters* **36**, 841-843 (2015).
20. Pyo, S.W. et al. An organic electrophosphorescent device driven by all-organic thin-film transistor using photoacryl as a gate insulator. *Current Applied Physics* **2**, 417-419 (2002).
21. Ryu, G. S. et al. Array of organic thin film transistors integrated with organic light emitting diodes on a plastic substrate. *Thin Solid Films* **514**, 302-305 (2006).

22. Yagi, I. et al. A Full-Color, Top-Emission AM-OLED Display Driven by OTFTs. *SID Symposium Digest of Technical Papers* **38**, 1753-1756 (2007).
23. Nakajima, Y. et al. Fabrication of 5.8 - in. OTFT - driven flexible color AMOLED display using dual protection scheme for organic semiconductor patterning. *Journal of the Society for Information Display* **17**, 629-634 (2009).
24. Galkin, A. L. et al. Relativistic motion and radiation of an electron in the field of an intense laser pulse. *Quantum Electronics* **37**, 903-909 (2007).
25. Choi, D.K. et al. Highly efficient, heat dissipating, stretchable organic light-emitting diodes based on a MoO_3/Au/MoO_3 electrode with encapsulation. *Nature Communications* **12**, 2864 (2021).
26. Hang, R. et al. High-performance, ultrathin, ultraflexible organic thin-film transistor array via solution process. *Small* **14**, 1801020 (2018).
27. Netzer, L. et al. Adsorbed monolayers versus Langmuir-Blodgett monolayers-Why and how? II: Characterization of built-up films constructed by stepwise adsorption of individual monolayers. *Thin Solid Films* **100**, 67-76 (1983).
28. Wang, S. et al. Directly Spin Coating a Low-Viscosity Organic Semiconductor Solution onto Hydrophobic Surfaces: Toward High-Performance Solution-Processable Organic Transistors. Advanced Materials Interfaces **7**, 1901950 (2020).
29. Wang, Y. Lieberman, M. Growth of ultrasmooth octadecyltrichlorosilane self-assembled monolayers on SiO_2. *Langmuir* **19**, 1159-1167 (2003).
